# Account of a Fatal Case of Apoplexy, with Particulars of the Dissection

**Published:** 1814-02

**Authors:** 


### For the Medical and Physical Journal.

### Account of a fatal Case of Apoplexy, with Particulars of the Dissection.

ABOUT nine in the morning I was sent for to visit an elderly person (between the ages of fifty and sixty), laboring under an attack of apoplexy, with symptoms particularly marked. The countenance was highly distorted; the eyes bloodshot; pupils much dilated, insensible to light, and drawn inwards; the breathing stertorous; the pulse quick, full, and hard. There was no time to be lost, and I immediately abstracted eighteen ounces of blood from the temporal artery, with surprising relief to the patient. The violently convulsive efforts subsided, the distortion of the countenance wore off, the eyes assumed their natural appearance, the breathing became free, and the pulse considerably slower, and much satisfaction was expressed at the amendment which had taken place. I left her, and rode forward to visit some patients in a neighbouring village. On my return, I was much gratified to find that her improvement had been progressive. A purgative glyster had procured two evacuations; and her intellectual faculties had in some measure resumed their functions. From these favorable appearances (notwithstanding she had already experienced three or four attacks of the same kind) my hopes of her recovery were very sanguine. These expectations, however, were completely dissipated at my next visit, which was on the following morning. The pulse was considerably quickened, and the tongue had assumed that dry appearance which attends high inflammatory action. As the pulse still possessed some degree of strength, I ventured to take eight ounces more blood from the arm, and directed a large blister to be applied between the shoulders: this, however, afforded little or no relief. Delirium came on, attended with insatiable thirst. She lingered till the fourth day, when death closed the scene. With some difficulty I obtained permission to open the head after death, and felt amply rewarded for my trouble by the satisfaction I experienced in discovering the immediate cause of all the alarming symptoms I had witnessed.

On attempting to remove the cranium after having sawed completely through the bone, I found it strongly adhered to the dura mater. The latter presented no unusual appearance, except a general fullness and distension of its vessels. The arachnoid tunic was perfectly natural, and nothing as yet discovered itself which could at all account for the violence of the symptoms; and although the sinuses of the dura mater were remarkably distended by a thick coagulum, gulum, I could discover no appearance of effusion. Upon a still more minute examination, however, I observed beneath the pia mater (which had a general blush of inflammation) a small dark spot, which immediately led to the seat of the complaint, by a careful dissection. I exposed a cavity situated in the posterior lobe of the left cerebrum, containing about two ounces of a dark-colored serum, tinged with blood. The sides of the cavity were perfectly smooth and vascular; and although I at first expected it, no communication whatever could be traced with either of the ventricles. I was now satisfied that I had discovered the source of all the mischief: curiosity, however, induced me (although pressed for time) to continue my dissection throughout the brain, the whole of which had a natural appearance, except an unusual turgescence of the vessels of the side affected, particularly of the choroid plexus.

On reviewing this case, the good effects of blood-letting are remarkably conspicuous, and afford every reason to believe that the patient's life would have been prolonged had not the disorder been so far advanced. P.

*Dulwich, Dec.* 14, 1813.

